# Factors affecting exclusive breastfeeding in the first month of life among Amazonian children

**DOI:** 10.1371/journal.pone.0219801

**Published:** 2019-07-11

**Authors:** Paola Soledad Mosquera, Bárbara H. Lourenço, Suely G. A. Gimeno, Maíra B. Malta, Marcia C. Castro, Marly Augusto Cardoso, for the MINA-Brazil Working Group

**Affiliations:** 1 Department of Nutrition, School of Public Health, University of São Paulo, São Paulo, Brazil; 2 Department of Global Health and Population, Harvard T.H. Chan School of Public Health, Boston, MA, United States of Ameirca; SUNY Upstate Medical University, UNITED STATES

## Abstract

Early life feeding practices can directly affect the growth, development, and survival of a child. This study aimed to estimate the frequency of and factors associated with exclusive breastfeeding (EBF) in the first month of life among Amazonian infants. We used data of 1,523 mother-child pairs of the MINA-Brazil birth cohort study. Mothers were interviewed soon after delivery at baseline and by telephone at 30–45 days postpartum (n = 962, 63.2% of those eligible). Kaplan-Meier survival analysis and accelerated failure-time (AFT) models were used to estimate the probability of EBF and the factors associated with EBF duration in the first month. At 30 days of age, 36.7% of the studied population (95% confidence interval [CI] 33.6–39.8) were exclusively breastfed, with a median duration of 16 days. Considering all eligible children for follow-up, the probability of EBF in the first month was 43.7% (95% CI 40.4–46.8), and the median duration was 30 days. The duration of EBF (time-ratio, TR) was 28% longer among multiparous mothers (TR 1.28; 95% CI 1.11–1.48). The use of a pacifier and the occurrence of wheezing were associated with a reduced EBF duration by 33% (TR 0.67; 95% CI 0.58–0.77) and 19% (TR 0.80; 95% CI 0.70–0.93), respectively. These results highlight that EBF among children in the Brazilian Amazon is considerably below international recommendations, and indicate the immediate need to plan and implement actions to promote and support breastfeeding early in life.

## Introduction

Good nutrition, healthcare, and sanitation during the first 1,000 days of life are essential to a child’s optimum health, growth, and neurodevelopment and provide lasting benefits throughout their life [1]. It is known that breast milk (BM) is the optimal feeding option for infants [2]. Besides meeting an infant’s nutritional needs, breastfeeding reduces mortality, morbidity and hospitalization from gastrointestinal and respiratory infectious diseases [3–5]. It is also inversely associated with overweight and obesity [6], high systolic blood pressure [7], and type I and type II diabetes [8,9], whereas it is positively associated with improved performance in intelligence tests in childhood and adolescence [10]. Additionally, increased breastfeeding benefits the health of the mother with longer periods of amenorrhoea, lower rates of breast and ovarian cancer and type II diabetes [11,12]. Considering the positive impacts breastfeeding has on health, the World Health Organization (WHO) recommends exclusive breastfeeding (EBF) for the first 6 months of life, and continued breastfeeding for up to 2 years or more [13].

Despite the scientific evidence that breastfeeding is superior over other forms of infant feeding, only 39% of children younger than 6 months worldwide are exclusively breastfed [14]. Although Brazil has seen substantial progress in the last decades, increasing more than tenfold the percentage of infants under 6 months exclusively breastfed in the last decades [15], the 2013 National Health Survey revealed that only 36.6% of infants younger than 6 months were exclusively breastfed [16]. According to the Second Survey on the Prevalence of Breastfeeding performed in 2008, and restricted to the Brazilian state capitals and the Federal District, the Northeast region had the lowest prevalence (37%) while the North region had the highest (46%); however, both numbers are far from the WHO’s recommendations [17].

Since EBF associated factors are multiple and dependent on the population studied [18], several epidemiological studies have described their magnitude and direction. Higher maternal schooling, multiparity, giving birth in a Baby Friendly Hospital–centres of BF support [19], and mother’s partner appreciation for breastfeeding are some factors related to increased EBF duration, whereas mother smoking during pregnancy, cracked nipples in the first month and pacifier use are associated with discontinuation of EBF [18,20–22]. Yet, few studies have explored the barriers to EBF in the first month of life, during which breastfeeding practices are being established. In addition, as confirmed by a recent systematic review from Brazil, most studies examining rates and associations with breastfeeding outcomes were based on cross-sectional surveys. Out of 27 studies on factors influencing EBF, only one-fourth of the studies used longitudinal data [23]. However, cross-sectional studies generally applying the 24-h recall method to gather information on infant feeding practices over-estimates EBF rates, whereas cohort studies based on the recall-since-birth method and reiterated measurements lead to more accurate estimates [24]. Also, in large countries such as Brazil, regional differences in infant feeding practices should be considered but are often poorly characterized. In the abovementioned systematic review, only one study from the North region, where the Brazilian Amazonian area is located, was included. Early consumption of foods other than BM in the first 6 months is strongly influenced by these regional differences [25]. This highlights the need to conduct analyses in different regions, population groups, and contexts in order to consistently protect, promote and support breastfeeding practices [26].

This study acknowledged the importance of breastfeeding for maternal and child health and the lack of data concerning factors affecting EBF in the Amazonian population. Therefore, we analysed data from the MINA-Brazil study (Maternal and Child Health and Nutrition in Acre, Brazil), the first population-based birth cohort conducted in the Brazilian Amazon, to estimate the frequency of EBF at 30 days, and to identify factors associated with EBF duration in the first month of life.

## Material and methods

### Study design and population

This study is part of the MINA-Brazil study, a population-based birth cohort in Cruzeiro do Sul, Acre State, Western Brazilian Amazon. In 2015, the city had 82,075 inhabitants of which 71.8% lived in the urban area. Participants were recruited between July 1, 2015 and June 30, 2016 at the Women’s and Children’s Hospital of Juruá Valley, the only maternity hospital in the region where 96% of the deliveries take place. All delivery-related admissions of women living in Cruzeiro do Sul were identified through daily visits by research team members. Within the first 12 hours after delivery, the study protocol was explained, and mothers were invited for participation, and baseline data was collected upon acceptance, as described elsewhere [27]. For the present study, inclusion criteria were singleton live births without any contraindication for breastfeeding [28] and fixed residence in Cruzeiro do Sul.

Written informed consent for participation was obtained from mothers before enrolment. For adolescent mothers, consent was given by their caretaker. This study was approved by the ethical review board of the School of Public Health, University of São Paulo, Brazil.

### Data collection and measures

The outcome of interest was the duration of EBF in the first month of life. According to the WHO definitions, EBF was classified as the intake of breast milk (direct from the breast, expressed, or from a wet nurse) without any additional liquids or solid/semi-solid foods except for oral rehydration salts (ORS), vitamins, minerals, and medications [29]. EBF was measured in the follow-up interview by asking mothers about the timing of introduction of liquids, semi-solid and solid foods from birth. We used the continuous variable expressed in days of exclusive breastfeeding and dichotomous (no or yes). Exposure to EBF considered socio-demographic characteristics, perinatal variables, and maternal-child factors during the postnatal period.

At the maternity hospital, trained research team members performed structured face-to-face interviews with each mother before hospital discharge. Information on area of residence (rural or urban), maternal age at delivery (<19 or ≥19 years), maternal education (<9 or ≥9 years of schooling), self-reported skin colour (white, black, brown, indigenous, or yellow as defined by the Demographic Census of the Brazilian Institute of Geography and Statistics), living with a partner (no or yes), parity (primiparous or multiparous), and presence of selected household assets was collected. As a proxy of the socioeconomic status, a wealth index based on standardized scores for household assets was generated with principal component analysis, as previously described [30].

Data on the number of antenatal care visits (<6 or ≥6 visits), type of delivery (vaginal or caesarean), child’s sex (female or male), birth weight (<2500 g, 2500–4000 g or ≥4000 g), gestational age at delivery in weeks (<37, 37–42 or ≥42 weeks), and breastfeeding initiation within the first hour of life (no or yes) were obtained from hospital records.

At 30–45 days postpartum, mothers were interviewed using a standardized telephone protocol, and information on the infant’s health conditions, such as diarrhoea, fever, cough, wheezing, ear problems, dengue fever, and hospitalizations in the first month of life (no or yes); current breastfeeding practice (no or yes); child’s age in days at introduction of weaning foods (cow milk and others, formula, water, tea, fruit juice, other liquids, semi-solids and solid foods); and use of a pacifier (no or yes) was collected. Furthermore, mothers were queried about smoking (no or yes), presence of cracked nipples or sore breasts (no or yes), and their perception of social support according to the Medical Outcomes Study survey, which contains 19 functional support questions and encompasses five dimensions of support: emotional, informational, tangible, affectionate, and positive social interaction [31]. For the present study, an estimated index of overall support was computed based on how often each type of support was available to mothers when required. To calculate the score, points were given to every response option for the 19 questions, ranging from zero (never) to four (always). The points of the answers given were added and divided by the maximum number of points possible to be reached. The score was then transformed so that the lowest possible score was 0 and the highest 100. Based on the median values of each dimension [32], the overall score was divided into 2 categories: low (<80) and high (≥80).

### Data quality control

Baseline data were entered into tablets using the Census and Survey Processing System (CSPro, U.S. Census Bureau, ICF International). Supervisors routinely checked all information and gave feedback to research team members to correct inconsistencies whenever necessary. Follow-up interviews were repeated in a random sample of 10% of study participants for quality control checks. Kappa and intraclass correlation coefficients for categorical and continuous variables, respectively, were calculated to analyse the reliability of the responses given by telephone to some variables of interest to classify exclusive breastfeeding practice. Overall, the coefficients in reported practices were in good agreement: 1.00 for current breastfeeding practice, 0.86 for tea consumption, 0.83 for other milk consumption, and 0.77 for the age of water introduction.

### Statistical analysis

Participants’ characteristics were described using means (standard deviations, SD) for continuous variables, and proportions (%) for categorical variables, and then compared according to categories of EBF practice at 30 days using Student's *t* test, *X*^*2*^ test or Fisher test.

Kaplan-Meier survival analysis was performed to estimate the median duration and probability of EBF at the end of the first month of life, allowing for the inclusion of all eligible infants. Those who were still exclusively breastfed at 30 days or participants who were lost to follow-up were considered censored observations. Interruption of EBF in the study period was treated as a failure.

Based on the Schoenfeld’s global test, the proportional hazards assumption was not valid for Cox models, so accelerated failure-time (AFT) models were performed in order to explore factors associated with EBF duration. In addition, Weibull distribution was found to be the best fit for the data according to Cox-Snell Residuals for different distributions (Exponential, Log-logistic and Log-normal) and the Akaike’s Information Criterion (AIC). Time ratios with 95% confidence intervals (95% CI) were used to interpret whether the factors increased or decreased EBF duration in the first month of life. Finally, a multiple adjusted model was fitted following a conceptual framework for hierarchical selection of covariates, in accordance with previous publications [23,33]. Missing observations were included by creating missing-value categories [34]. In addition, Pearson’s correlation test was assessed to evaluate the existence of linear dependence between variables tested in the regression model and EBF duration. All analyses were conducted in Stata version 15.0 (StataCorp, College Station, TX, USA), with significance set at p<0.05.

## Results

**Fig 1:**
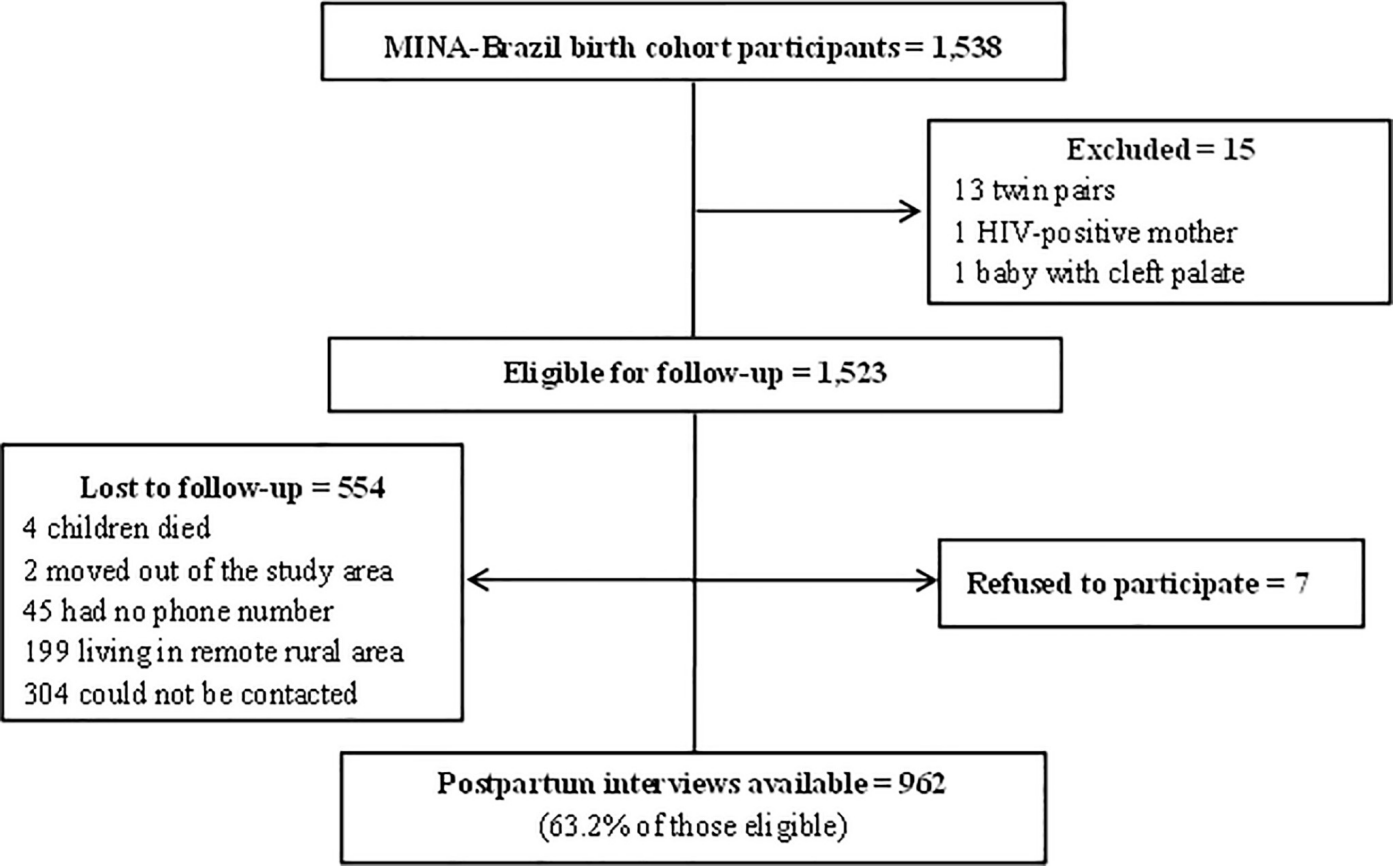
Flow chart of participants in the MINA-Brazil birth cohort study for analysis of exclusive breastfeeding practice during the first month of life.

Overall, 1,538 participants were enrolled at baseline in the MINA-Brazil birth cohort, of which 1,523 participants were considered eligible for the present analysis. We excluded 13 mothers who had multiparity births, 1 HIV-positive mother, and 1 mother who had a baby with cleft palate. During follow-up, 4 children died, 2 mother-child pairs moved out of the study area, 45 mothers had not provided a valid telephone number, 503 mothers could not be reached after several contact attempts. In addition, 7 (0.5%) mothers declined participation at the assessment. Thus, information at 30–45 days postpartum was available for 962 (63.2% of those eligible for follow-up) mother-child pairs (Fig 1). The median postpartum time elapsed from delivery until the follow-up interview was 37 days. When comparing the baseline characteristics of the mothers contacted at follow-up (n = 962) with participants lost to follow-up from the urban area (n = 322), no significant differences were observed. However, when comparing the baseline characteristics of the contacted mothers with participants lost to follow-up living in the rural area (n = 239), high frequencies of mothers with less than 9 years of schooling (36.4% vs. 74.2%), mothers belonging to the first quintile of wealth (13.8% vs. 55.7%), and mothers living with their partners (75.8% vs. 83.4%) were found, respectively.

### Participants’ characteristics

Characteristics of the study participants are summarized in Table 1. Mean (SD) maternal age was 25 (6.6) years, 63.5% of mothers had 9 or more years of formal education and 78.9% of mothers reported their skin colour as brown. Moreover, 47.4% of mothers were primiparous, and 75.4% attended at least six antenatal care visits. Roughly half of deliveries were vaginal (54.4%). Most infants were born within 37–42 weeks gestational age range (90.7%), and breastfed within the first hour of life (88.5%).

**Table 1:** Characteristics of the mother-child pairs participating in the MINA-Brazil birth cohort. Cruzeiro do Sul, Acre. 2015–2016 (n = 962).

Variables (n)^a^	Categories	n (%)
**Maternal age at delivery (years)**	< 19	187 (19.5)
	≥ 19	775 (80.5)
**Self-reported skin color (925)**	White	108 (11.7)
	Black	32 (3.5)
	Brown	730 (78.9)
	Indigenous	55 (5.9)
**Maternal education, years (925)**	0 |-| 9	337 (36.5)
	> 9	588 (63.5)
**Household wealth index, quintiles (925)**	1^st^ lower	128 (13.8)
	2^nd^	178 (19.2)
	3^rd^	196 (21.2)
	4^th^	214 (23.1)
	5^th^ higher	209 (22.6)
**Mother living with her partner (925)**		701 (75.8)
**Area of residence**	Urban	871 (90.5)
	Rural	91 (9.5)
**Parity (925)**	Primiparous	438 (47.4)
	Multiparous	487 (52.6)
**Antenatal care visits (960)**	< 6	236 (24.6)
	≥ 6	724 (75.4)
**Type of delivery**	Vaginal	523 (54.4)
	Cesarean	439 (45.6)
**Child’s sex**	Female	480 (49.9)
	Male	482 (50.1)
**Gestational age**	< 37	73 (7.6)
	37 |- 42	873 (90.7)
	≥ 42	16 (1.7)
**Birth weight (grams)**	< 2500	63 (6.5)
	2500 |- 4000	844 (87.7)
	≥ 4000	55 (5.7)
**Breastfeeding within the first hour (907)**		803 (88.5)
**Breastfeeding practice in the first month**	Exclusive	353 (36.7)
	Non-exclusive	594 (61.7)
	Not-breastfed	15 (1.6)
**Breast problems**^**b**^ **(958)**		545 (56.9)
**Smoking in the postpartum period (958)**		25 (2.6)
**Social support—*total score* (946)**	Low	458 (48.4)
	High	488 (51.6)
**Use of pacifier (960)**		276 (28.7)
**Reported infant health conditions**	Diarrhea	103 (10.7)
	Fecal blood	24 (2.5)
	Fever	186 (19.3)
	Wheezing	312 (32.4)
	Dry cough	106 (11.0)
	Catarrh	102 (10.6)
	Ear problems	23 (2.4)
**Child’s hospitalization (960)**		40 (4.1)

^a^ Totals may differ due to missing values.

^b^ Sore breasts, cracked nipples or both.

A predominant proportion of children (63.3%) were exposed to other foods or liquids other than BM in the first month including tea (38.8%), water (31.4%), non-human milk and formula (30.7%), and pounded cassava (10.3%). For 2.2% of the babies, coconut water, fruit juices, porridge, yogurt, condensed milk, and rice porridge had already been offered by 30 days of age (data not shown in Tables). Consequently, although 98.4% of the studied babies were still breastfed at the end of the first month of life, only 36.7% received EBF, with a median duration of 16 days.

### Probability of EBF and factors affecting its duration

**Fig 2:**
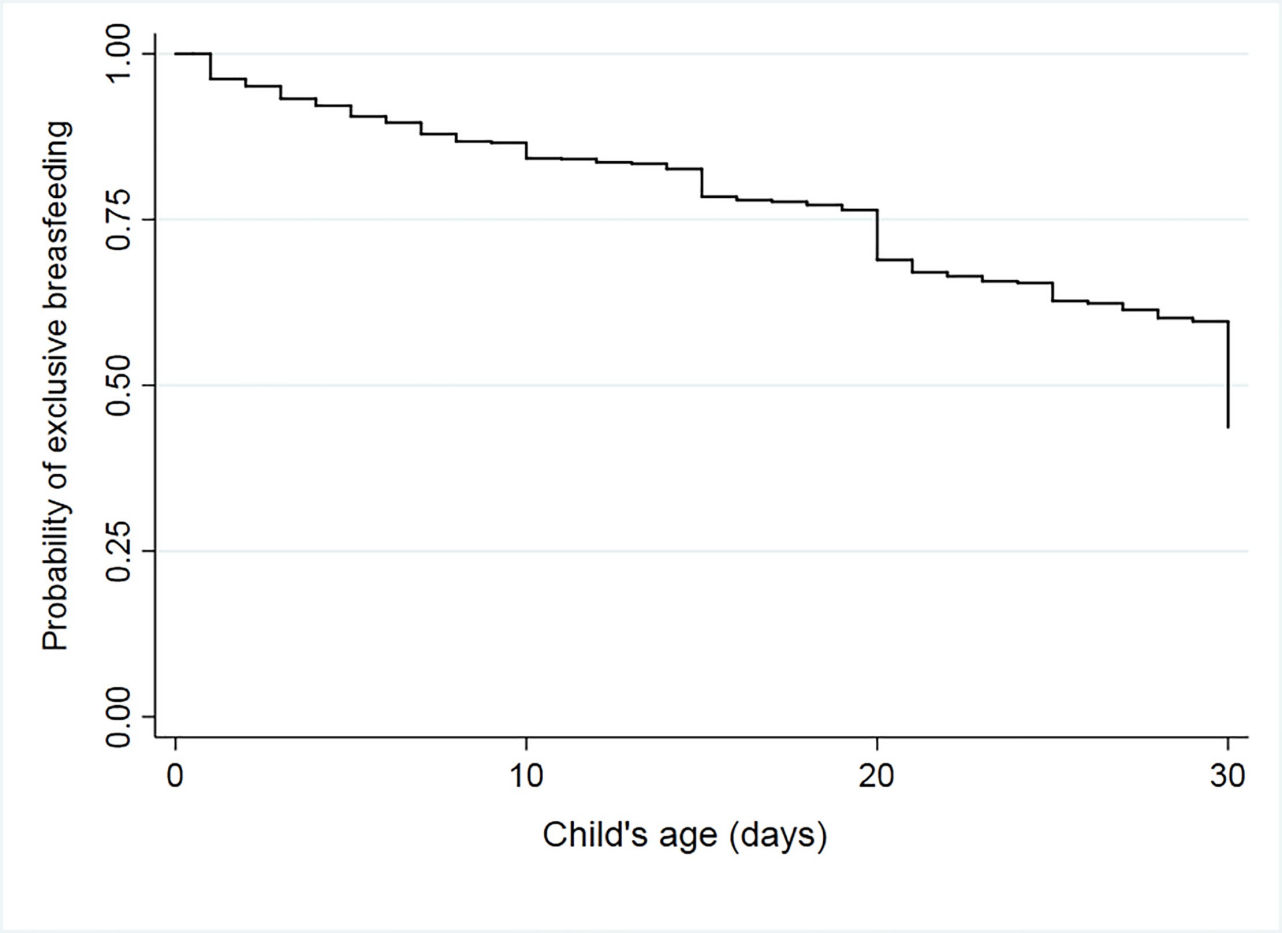
Kaplan-Meier survival curve for exclusive breastfeeding at 30 days of age in the MINA-Brazil birth cohort study.

Fig 2 shows the Kaplan-Meier survival curve for the probability of EBF in the first month of life. Considering all eligible children for follow-up, the probability of infants being exclusively breastfed in the first day was 96.2% (95% CI 95.1–97.0), decreasing to 75% by day 19 (95% CI 74.1–78.6). At 30 days, the probability of survival of EBF was only 43.7% (95% CI 40.4–46.8). The median duration of EBF was 30 days, at which time there was a significant decrease (16%) in infants who continued to receive only BM on the following day.

In the crude analysis, mother living with her partner, living in the rural area and having one or more children were positively associated with a higher probability of EBF. Conversely, mother aged <19 years, presenting breast problems, using pacifier, and with occurrence of morbidities, such as diarrhoea, wheezing, and fever, were related to a lower probability of EBF. However, there were no significant associations between EBF and other variables, including maternal education, self-reported skin color, household wealth index, antenatal care visits, type of delivery, child’s sex, gestational age, birth weight, breastfeeding within the first hour, smoking in the postpartum period, social support, infant health conditions such as fecal blood, dry cough, catarrh, ear problems and child’s hospitalization. Variables associated with EBF in the crude analyses were included in AFT models with adjustment for the child’s sex, gestational age, type of delivery, breastfeeding in the first hour of life, and household wealth index. Three variables remained consistently associated with EBF duration in the first month of life: multiparous mothers, use of pacifier, and the occurrence of wheezing. Compared to primiparous mothers, EBF duration was 28% longer among multiparous mothers, whereas the use of a pacifier and the occurrence of wheezing reduced the EBF duration by 33% and 19%, respectively (Table 2). Indeed, multiparous mothers (r = 0.0532), use of pacifier (r = -0.2020), and wheezing (r = -0.092) were significantly correlated with EBF duration (p<0.05; S1 Fig).

**Table 2:** Factors associated with time of exclusive breastfeeding duration in the first month of life in the MINA-Brazil birth cohort study.

Variable	HR^a^ (95%CI)* Crude analysis	TR^b^ (95%CI)* Adjusted model
**Maternal age at delivery (years)**		
≥ 19	1	
< 19	1.27 (1.04–1.53)	-
**Mother living with her partner**		
No	1	
Yes	0.77 (0.64–0.94)	-
**Area of residence**		
Urban	1	
Rural	0.52 (0.39–0.67)	-
**Parity**		
Primiparous	1	1
Multiparous	0.67 (0.57–0.79)	1.28 (1.11–1.48)
**Breast problems**^**c**^		
No	1	
Yes	1.24 (1.05–1.47)	-
**Use of pacifier**		
No	1	1
Yes	1.65 (1.39–1.96)	0.67 (0.58–0.77)
**Diarrhoea**		
No	1	
Yes	1.35 (1.05–1.71)	-
**Wheezing**		
No	1	1
Yes	1.33 (1.12–1.57)	0.81 (0.70–0.93)
**Fever**		
No	1	
Yes	1.25 (1.03–1.53)	-

^a^Hazard ratio.

^b^Time Ratio.

^c^Sore breasts, cracked nipples or both

*p<0.05

**Fig 3:**
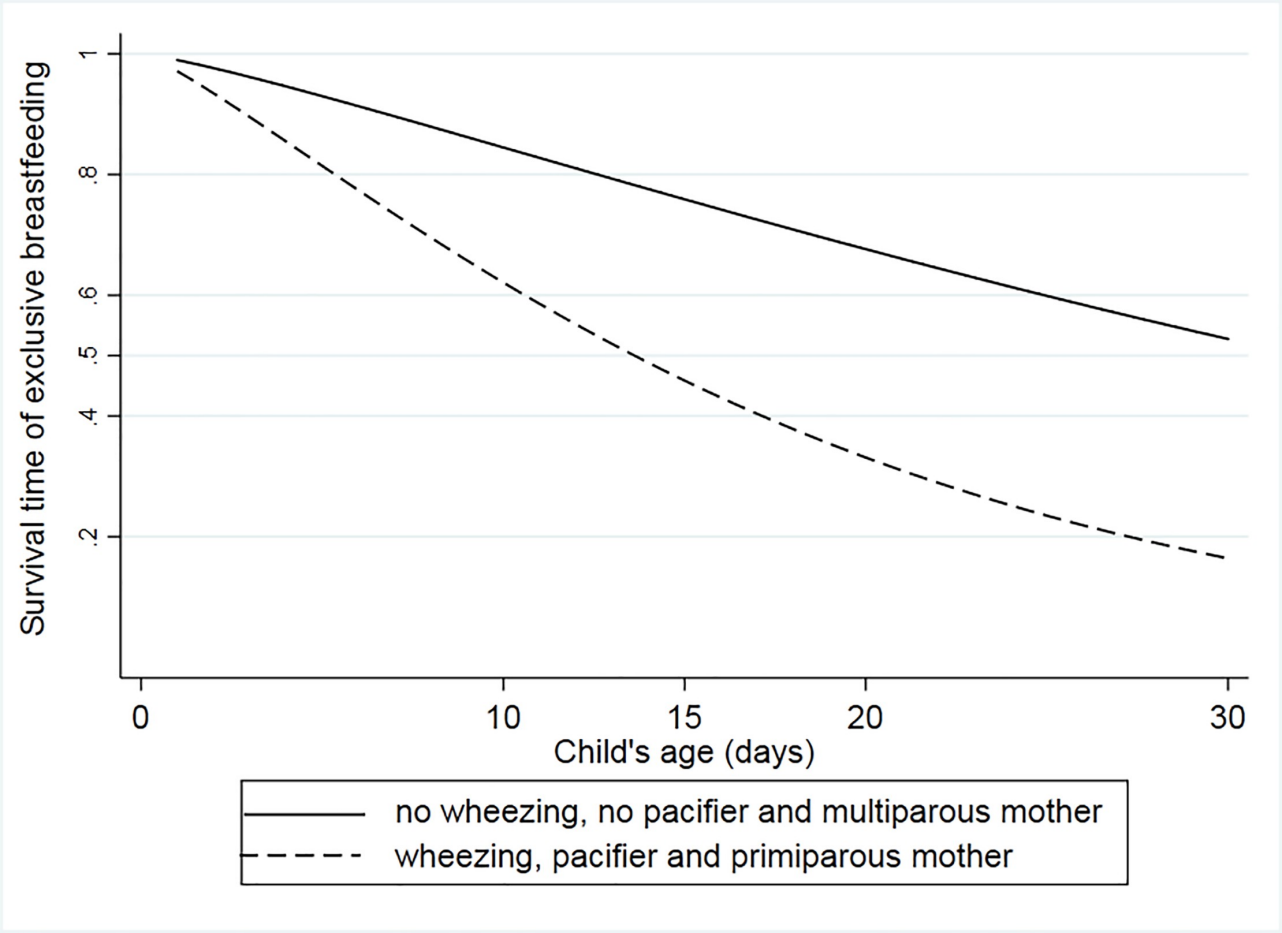
Survival function of time to cessation of EBF in the first month of age, adjusted for parity, use of pacifier and occurrence of wheezing in the MINA-Brazil birth cohort study.

Fig 3 shows the survival function of time to cessation of EBF in the first month of age, considering the final adjusted model. The upper curve represents a child most likely to be exclusively breastfed for longer in the first month of life. Characteristics of that child include a multiparous mother, non-exposure to a pacifier, and no previous wheezing episodes (28% of the study population fits this profile). In comparison, a child with the opposite characteristics and consequently, with an increased risk of early EBF interruption, is represented by the lower curve (6% of the study population fits this profile). Under these conditions, slightly over 50% of children represented in the upper curve versus less than 20% in the lower curve would maintain EBF at 30 days of life.

## Discussion

Based on data from the first population-based birth cohort study in the Western Brazilian Amazon, we found that more than half of the infants had interrupted EBF before the first month of life. For those who had stopped EBF, the median EBF duration was only 16 days. Factors associated with a lower duration of EBF in the first month of life were primiparity, use of a pacifier, and occurrence of wheezing.

In this study, the percentage of studied children exclusively breastfed at 30 days of age (36.7%) was far from compliant with the WHO recommendation of EBF in the first six months of life [13]. In addition, this number is much lower than what was previously reported from prospective studies conducted in high-income countries including England (52.1%) [35], Spain (76.2%) [36], Italy (61.2%) [37] and Greece (43.5%) [38]. Such differences might be explained by the fact that wealthier mothers generally have higher educational level. Increased education often enables mothers to make wise decisions regarding child feeding practices [39]. Thus, a higher maternal education level, usually used as a proxy for economic status, may be a good predictor of successful EBF practice [21,40]. In our study, a higher proportion of exclusively breastfed babies was found among mothers with more than nine years of schooling. However, this characteristic did not significantly affect EBF. This result may be related to underlying factors that could influence the relationship between personal attributes and EBF rates, such as access to health care services and social, cultural and market context [41], which we did not investigate. Further research into these factors in different population settings is needed.

Among low-middle income settings, a prospective analysis of pooled data from three randomised trials performed in Ghana, India, and Tanzania (60.1%), reported greater EBF estimates at 30 days [42]. Similarly, a prospective population-based cohort conducted in Pakistan (75.9%), India (99%), Kenya (85.5%), Zambia (98.7%) and Guatemala (84.6%) also described higher EBF rates at 42 days [43]. These differences were possibly due to a different methodological design, classification of EBF, and sociocultural aspects among the studied population. Nevertheless, our result was equivalent to that found at 30 days in a prospective community-based study performed in the city of Loreto (38.2%), located in the Peruvian Amazon [44]. Likewise, it matched the Brazilian average of 36.6% for EBF up to 6 months [16], yet it was also much smaller than previous longitudinal studies using data from Brazil that reported percentages of EBF in the first month of age ranging from 77% to 90% [18,45]. This discrepancy suggests that Amazonian infants were exposed to a lower chance of being exclusively breastfeed and, thus, were at high risk for foodborne diseases, childhood malnutrition and neonatal mortality [13], which would constitute a relevant public health challenge.

In such scenario, the importance of the Baby Friendly Hospital (BFH) initiative and its ten steps to successful breastfeeding must be acknowledged [46]. In the study area, the reference maternity hospital offers beds for rooming-in and the kangaroo method, a weekly Shantala technique course [47] with a human milk bank available; however, it is not yet certified as a BFH. Thus, by increasing systematic EBF interventions, including individual counselling, group education, immediate support for postnatal breastfeeding, and lactation management, the observed EBF rates might be improved [41]. In this respect, despite the high frequency of antenatal care visits and breastfeeding in the first hour of life found in this study, the high percentage of mothers reporting breast problems (56.9%) might indicate that little support is being offered to women in order to establish breastfeeding and bring attention to the gap in the local health service. According to previous studies in Brazil [23], other contextual factors influencing EBF rates should also be considered such as norms and attitudes towards breastfeeding, level of acceptance of breastfeeding promotion, and protection and support policies and actions. Further studies as well as greater efforts should be made in this population in order to better understand the factors affecting early EBF rates.

In our study, EBF duration was 28% higher among mothers who were not primiparous, a result previously observed in both developed [21,36,40] and developing countries [43]. Primiparous mothers may tend to not offer BM to their offspring, especially those younger than 4 and 6 months of age under EBF [48]. However, other Brazilian cohort studies did not observe an association between parity and EBF [18,49,50]. According to Vieira et al. (2004) [51], cultural practices of early introduction of teas, water, and foods along with BM, probably have a greater impact on practices with the first child when mothers are not well supported for exclusive breastfeeding. Thus, mothers with previous experience in the breastfeeding process and possibly older, are usually more mature in what concerns care and feeding of a child when compared to primiparous women.

The use of pacifiers negatively affected the duration of EBF, which was widely corroborated in studies conducted worldwide. In Brazil, cross-sectional and cohort studies indicated that exposure to pacifiers was the most strong proximal factor associated with discontinuation of EBF [23]. Our study also found such an association: three-fourths of the babies who used a pacifier were no longer exclusively breastfed at the end of the first month of life, and among them, EBF duration was 33% lower. Similarly, in a cohort study performed in Bahia, Brazil, Vieira et al. [18] identified a 40% higher risk of EBF cessation before 6 months of age among infants who used a pacifier. This inverse association has also been reported in longitudinal investigations in Italy [52], Australia [22], and China [53]. While the specific mechanisms related to the association between pacifier use and reduced breastfeeding are not fully understood, the use of pacifiers may interfere with the breast suction technique [54]; may impair BM production due to a reduced frequency of breastfeeds [55]; or could be a proxy for bottle feeding or of mother’s little interest in breastfeeding [56]. Regardless of the mechanism, the evidence shows that infants using pacifiers present a greater risk of not receiving the benefits of breastfeeding in early life [20].

We observed early introduction of liquids or solids in a predominant percentage of children (63.3%). The protective effects of BM against infectious diseases can be substantially reduced when the child receives other foods, including tea and water, in addition to BM before six months of age [2], and such association has been observed in Brazil [5,57] and other countries [4,58]. In our study, occurrence of a wheezing condition during the first month of life was associated with a lower duration of EBF. Thus, we might infer that the early interruption of EBF may have contributed to wheezing episodes, since EBF for at least 3 months was found to be an effective practice to postpone the first wheeze episode in the first year of life in Latin American countries [59]. Conversely, our result could be interpreted as wheeze disorder leading to lower duration of EBF. This association had not yet been described, although in developed countries and among high-risk populations (infant born to mothers with a history of asthma) there have been reports showing that the onset of eczema or wheezy symptoms prolongs EBF duration due to its well-known protective effect [60,61]. However, the effect of early symptoms of atopic disease on breastfeeding duration in general birth cohort studies is still unclear [60]. Wheezing infants’ prevalence varies among settings, being more frequent in Latin American than in European countries [62]. This difference implies the influence of local environmental factors on the clinical expression of several wheezing phenotypes in childhood [63]. Thus, the prevalence of factors, such as maternal asthma, smoking, antibiotic use and caesarean sections, may influence the willingness and capacity of mothers to breastfeed [64]. In addition, this finding should be interpreted with caution mainly because data on wheezing episodes is based on maternal report without medical confirmation. It is likely that some women might have confused other frequent sounds of the upper respiratory tract with wheezing episodes. The result, therefore, may over-represent the actual association, whereas more specific information might have led to different findings. Further research with more accurate data is required in order to elucidate wheezing exposure relative to breastfeeding practice.

The findings described in our study are helpful in supporting the formulation of public health interventions to promote initiation and maintenance of exclusive breastfeeding. Health professionals should play a crucial role, from pregnancy to the postpartum period, in advising and managing breastfeeding problems. For instance, they could inform pregnant women on the effects of pacifier use on breastfeeding success and refer them to the maternity Shantala technique course [47], which includes BF counselling, as early as feasible after childbirth. Moreover, discussing the baby positioning and latch-on techniques, during the prenatal and postnatal period, may benefit mothers in the prevention of nipple pain and trauma [65]. However, given that health care support seems to be limited at the local level, peer support comes out as an indispensable strategy. Health professional and peer trained support, if coordinated and continuous from the beginning of pregnancy, may increase EBF rates [66].

Our study has some limitations. Although all eligible children were included in the survival analysis, losses to follow-up inherent to cohort studies implied a lack of information on EBF practices that may have influenced our outcomes. Moreover, data relied on self-reported information, such as infant’s health and morbidity characteristics, possibly leading to under or overestimation of frequencies. Lastly, the differences found between baseline characteristics of the mothers contacted at follow-up and the mothers living in the rural area lost to follow-up could have caused selection bias. Thus, the results presented in this study should not be generalized to all mothers, but more specific to the urban mothers. However, this study has several strengths. First, this is the first population-based birth cohort study in the Western Brazilian Amazon to estimate frequency of EBF at 30 days of life, and to identify factors associated with EBF duration in this time period. Second, since data on infant feeding practices were collected shortly after birth (30–45 days), maternal recall bias was likely small, increasing the reliability of our results. Third, the proportion of infants exclusively breastfed at 30 days was estimated using information on each child’s age at introduction of weaning foods, rather than based on direct maternal reports, thus avoiding possible misclassification. Fourth, considering that data collected from the hospital records are generally of poor quality [67], in what regards information on breastfeeding in the first hour of life, we confirmed that 94.3% of the children registered as "yes" for this practice were also breastfed on the first day, according to data collected by the research team members. Finally, we adhered to the most current definition of breastfeeding practices [29] to classify EBF from birth to the first month.

In conclusion, EBF rate at 30 days in Amazonian infants was below international recommendations. The duration of EBF in the first month of life was longer among multiparous mothers. In contrast, infants who used pacifiers or presented wheezing episodes had a shorter duration of EBF. The low rate of EBF in this study population coupled with an early introduction of complementary foods, raise concerns about the impact on maternal and child health. Thus, health professionals should target interventions to all the women in the study area, with special emphasis on mother-child pairs at a greater risk of discontinuing EBF, in order for them to benefit from an appropriate breastfeeding practice from the first days of life.

## Supporting information

S1 Fig Factors associated with EBF duration in the first month of life in the MINA-Brazil birth cohort study. (TIF)
