# Supplementary figures and images for: Factors affecting exclusive breastfeeding in the first month of life among Amazonian children
Source: PLoS One. 2019 Jul 11;14(7):e0219801. doi: 10.1371/journal.pone.0219801 (PMC6623463; doi:10.1371/journal.pone.0219801)

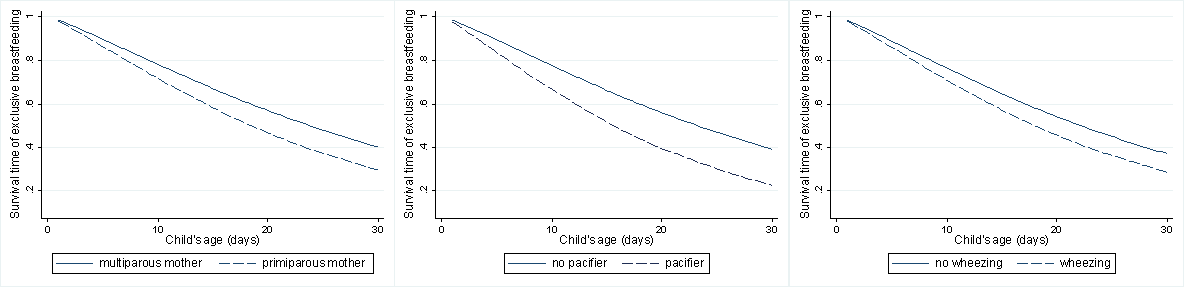

Supplement: S1 Fig — (TIF) [file pone.0219801.s001.tif]
